# Organocatalytic asymmetric synthesis of P-stereogenic molecules

**DOI:** 10.3389/fchem.2023.1132025

**Published:** 2023-02-16

**Authors:** Junyang Liu, Hang Chen, Min Wang, Wangjin He, Jia-Lei Yan

**Affiliations:** ^1^ Innovation Center of Marine Biotechnology and Pharmaceuticals, School of Biotechnology and Health Sciences, Wuyi University, Jiangmen, China; ^2^ State Key Laboratory of Chemical Oncogenomics, Key Laboratory of Chemical Genomics, Peking University Shenzhen Graduate School, Shenzhen, China; ^3^ Division of Chemistry and Biological Chemistry, School of Chemistry, Chemical Engineering and Biotechnology, Nanyang Technological University, Singapore, Singapore

**Keywords:** organocatalysis, asymmetric synthesis, desymmetrization, kinetic resolution, P-chirality

## Abstract

P-chirality broadly appears in natural and synthetic functional molecules. The catalytic synthesis of organophosphorus compounds bearing P-stereogenic centers is still challenging, due to the lack of efficient catalytic systems. This review summarizes the key achievements in organocatalytic methodologies for the synthesis of P-stereogenic molecules. Different catalytic systems are emphasized for each strategy class (desymmetrization, kinetic resolution, and dynamic kinetic resolution) with examples cited to illustrate the potential applications of the accessed P-stereogenic organophosphorus compounds.

## Introduction

**FIGURE 1:**
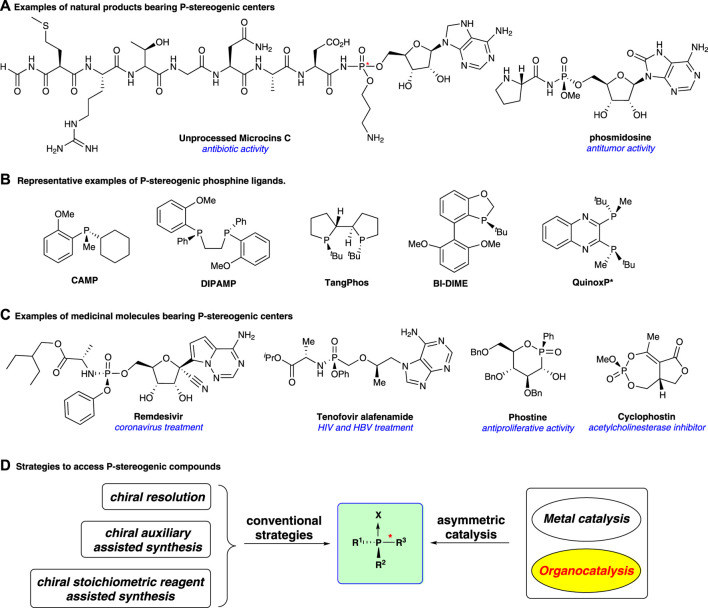
P-stereogenic compounds and the existing synthetic strategies. **(A)**: Examples of natural products; **(B)**: Examples of P-stereogenic phosphine ligands; **(C)**: Examples of medicinal molecules; **(D)**: Strategies to access P-stereogenic compounds.

Organophosphorus compounds bearing P-stereogenic centers have widely emerged in biological molecules and natural products (Figure 1A)(Kolodiazhnyi, 2021), and they also serve as broadly useful ligands and catalysts in asymmetric synthesis (Dutartre et al., 2016; Xu et al., 2019; Imamoto, 2021) (Figure 1B). Nowadays, P-stereogenic scaffolds show an increasing presence in bioactive molecules for medical uses (Figure 1C). For example, remdesivir is used to treat coronavirus disease (Wang et al., 2020a). Tenofovir alafenamide is an antiviral prescription medicine for the treatment of HIV (Ray et al., 2016) and chronic hepatitis B infection (Scott and Chan, 2017); phostine serves as an anti-malignant proliferation agent (Bousseau et al., 2019). Cyclophostin is an inhibitor of acetylcholinesterase (Martin et al., 2015). It is worth noting that the absolute stereochemistry of phosphorus is often directly associated with the biological activity of these molecules (Pradere et al., 2014; Nocentini et al., 2019; Babbs et al., 2020; Nocentini et al., 2020). Thus, developing efficient strategies to access P-stereogenic organophosphorus compounds is of great importance.

In the early years, optically pure P-stereogenic compounds were obtained by relying on the resolution of organophosphorus enantiomers or the related diastereomeric mixtures (Meisenheimer and Lichtenstadt, 1911). The pioneering asymmetric strategy to access P-stereogenic molecules is chiral auxiliary-assisted synthesis, in which the auxiliary is bound to the P-atom to control the stereochemistry (Farnham et al., 1970; Berger and Montchamp, 2013). Similarly, using stoichiometric chiral reagents to influence the enantiomeric outcome of the P-stereogenic center is also a viable approach (Muci et al., 1995; Bergin et al., 2007; Kortmann et al., 2014). However, stoichiometric amounts of chiral reagents are essential in all the aforementioned strategies. In parallel, catalytic asymmetric strategies to access P-stereogenic molecules are more succinct and economic. These catalytic strategies have had an impressive breakthrough in the past two decades, especially in transition metal catalytic systems (Lemouzy et al., 2020; Ye et al., 2021). In contrast, organocatalytic asymmetric strategies were not so developed until recent years. In order to guide a better understanding, this review will focus on organocatalytic strategies and introduces the most recent developments of stereoselective access to P-stereogenic compounds.

## Asymmetric desymmetrization strategies

**FIGURE 2:**
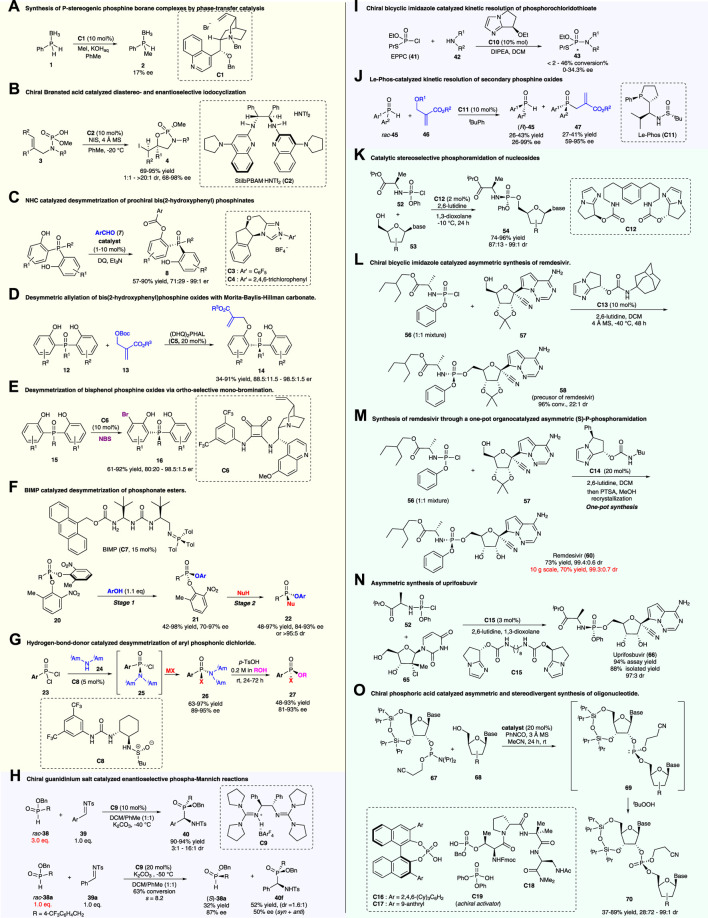
Organocatalytic asymmetric strategies for access to P-stereogenic compounds. **(A–G)**: Desymmetrization strategies; **(H–J)**: Kinetic resolution; **(K–O)**: Dynamic kinetic resolution strategies.

A powerful strategy to access P-stereogenic compounds is the desymmetrization of symmetrical achiral organophosphorus compounds, which has accounted for a large part of the catalytic synthesis of P-stereogenic compounds. This pioneering work was reported by Lebel et al. (2003), in which catalytic alkylation of phosphine–boranes for constructing P-stereogenic phosphine borane **2** was demonstrated using a *Cinchona* alkaloid-derived catalyst **C1** as a phase-transfer catalyst (Figure 2A; Supplementary Scheme S1). Although enantioselectivity was not satisfying (17% ee), this work greatly encouraged the synthesis of P-stereogenic phosphorus compounds through asymmetric organocatalysis.

In 2014, Johnston and co-workers reported a chiral Brønsted acid-catalyzed diastereo- and enantio-selective iodocyclization of phosphoramidic acid for the construction of C- and P-stereogenic cyclic phosphoramidates (Toda et al., 2014) (Figure 2B; Supplementary Scheme S2). Utilizing this strategy, a range of cyclic products (**4a**–**4f**) was prepared with high levels of absolute and relative stereocontrol (up to >20:1 dr, 98% ee). The resulting phosphoramidate products acted as precursors for enantio-enriched epoxy allylamines (**5**) upon treatment with alkoxy anions. Thus, this method could be regarded as a formal asymmetric epoxidation of allylamine derivatives.

With the diverse development of organocatalysis, more investigations on P-stereogenic constructions were performed. In 2016, the Chi group reported *N*-heterocyclic carbene (NHC)-catalyzed desymmetric acylation of pro-chiral bisphenol phosphine oxides for the synthesis of P-stereogenic phosphinates, phosphinamides, and triarylphosphine oxides (Figure 2C; Supplementary Scheme S3) (Huang et al., 2016). Good to excellent yields and enantioselectivities were realized in this work. Moreover, this reaction possesses a wide substrate scope and could be performed on a gram-scale with low catalyst loading (1 mol%). To further demonstrate the utility of this methodology, the P-stereogenic product **8a** was converted to the chiral bidentate Lewis base **10** and the precursor of the chiral ligand DiPAMP *via* simple transformations. The newly synthesized bidentate Lewis base **10** could be directly used as a catalyst in asymmetric reductive aldol reactions of enones and aldehydes.

Based on the bis(2-hydroxyphenyl) phosphine oxides, the Li group demonstrated a biscinchona alkaloid-catalyzed desymmetric allylation reaction with the Morita–Baylis–Hillman carbonate to produce P-stereogenic phosphine oxides (Figure 2D; Supplementary Scheme S4) (Yang et al., 2019). Multiple functional groups were tolerated under mild reaction conditions, with a wide range of chiral P-stereogenic phosphine oxides prepared with good yields (up to 99%) and high enantioselectivities (up to 98.5:1.5 er). Additionally, large-scale reactions and synthetic transformations were also conducted in the study. Mechanically, theoretical calculations revealed that the ΔΔG^‡^ value between **TS1** and **TS2** was 2.2 kcal mol^−1^ (Supplementary Scheme S4b), and the stabilization effect of the C–H ^…^ π interaction between the catalyst and substrate (as shown in **TS1**), as well as the destabilization steric effect of bulky *tert*-butyl (as shown in **TS2**), were the key factors that contributed to the energy difference of the two transition states, which were crucial for the excellent enantioselectivity control.

Recently, the Li group reported an alternative strategy for the desymmetrization of bisphenol phosphine oxides using chiral squaramide-catalyzed ortho-selective mono-bromination (Figure 2E; Supplementary Scheme S5) (Huang et al., 2021). This reaction could provide a series of chiral bisphenol phosphine oxides and phosphinates with good to excellent yields (up to 92%) and enantioselectivities (up to 98.5:1.5 er). Furthermore, this reaction could be scaled up to 1.0 mmol without the loss of the er value for **16a**. The *ortho*-brominated P-stereogenic product can be further transformed into functional molecules, which retained the optical purities, *via* reactions including metal-catalyzed cross-couplings, *O*-alkylations, or nucleophilic substitutions in P-centers (Supplementary Scheme S5b). This asymmetric *ortho*-bromination strategy provided an alternative route for the desymmetrization of bisphenol phosphine oxides.

In contrast to the desymmetric functionalization of bisphenol phosphine oxides, direct nucleophilic desymmetrization at the P-center, with the formation of a new P–X bond, is a more challenging but powerful strategy. In 2021, Dixon’s group published a preprint work, in which a novel bifunctional iminophosphorane (BIMP, **C7**) catalytic two-stage desymmetrization strategy for the construction of P-stereogenic compounds was reported (Figure 2F; Supplementary Scheme S6) (Formica et al., 2021). This process involves BIMP-catalyzed asymmetrically nucleophilic substitution of one phenolic leaving group at the P-center (first stage) and subsequent enantiospecific displacement of the other phenolic leaving group *via* SN2 substitution (second stage), which allows quick access to a diverse range of chiral P(V) compounds including those with *O*-, *N*-, and *S*-linkages. Notably, nucleophilic phenols with an *ortho*-substituent were essential for the first stage. Also, the *O*-, *S*-, and *N*-centered nucleophiles were all suitable for the second stage, which gave rise to a range of chiral phosphonate esters, phosphorothioates, and phosphonamidite esters, with good yields and high enantioselectivities or diastereoselectivities.

Pro-chiral phosphonic dichlorides are also suitable substrates for the acquisition of P-stereogenic molecules *via* asymmetric desymmetrization. Forbes and Jacobsen (2022) reported hydrogen bond donor **C8**-catalyzed desymmetrization of pro-chiral phosphonic dichloride *via* enantioselective substitutions at the P-center for the preparation of aryl chlorophosphonamidates, which were developed as versatile P(V)-stereogenic building blocks. After the first desymmetric substitution step, the remaining two leaving groups (chloro and amino groups) on chlorophosphonamidates (**25**) can be displaced sequentially and stereospecifically to give a diverse range of P(V)-stereogenic compounds through substitutions with different nucleophiles (e.g., alkoxides, phenoxides, thiolates, deprotonated carbamates, and Grignard reagents) (Figure 2G; Supplementary Scheme S7a). A series of P(V)-stereogenic compounds were obtained in good yields and high optical purities, except for reactions using alkyl phosphonic dichlorides as substrates. The phosphonamidite product **26** could further be converted to a wide range of phosphonates, phosphonate thioesters, phosphinates, and phosphonamidates with retained enantioselectivities or slight loss via acid-promoted nucleophilic substitution of the diisoamyl amino group (Supplementary Scheme S7b).

To further demonstrate the synthetic utility of this hydrogen bond donor catalytic strategy, Forbes and Jacobsen have achieved three-step synthesis of the utrophin modulator (+)-SMT022332 (**31**) and formal synthesis of a matrix metalloproteinase (MMP) inhibitor (**37**) (Supplementary Scheme S8). The subjection of phosphonic dichloride **28** to the optimized conditions for catalytic enantioselective substitution produced phosphonamidate **29** (68% yield; 95% ee). After sequential methanolysis and phenol displacement, phosphonamidate **30** was converted to (+)-SMT022332 (**31**) with 94% ee, 100% es, and 43% overall yield over the three steps. In the formal synthesis of the matrix metalloproteinase inhibitor (**37**), *N*-allyl benzylamine (**33**) was used in the substitution reaction of phosphonic dichloride **32** under modified conditions, with high enantioselectivity obtained as well. The subsequent ring closing-metathesis and related transformations generated the target MMP inhibitor (**37**). It is anticipated that *N*-allyl benzylamine’s versatility as a masked “–NH_2_” equivalent may enable access to a wide variety of other phosphonamidate targets.

## Asymmetric kinetic resolution strategies

The catalytic kinetic resolution of racemic P-stereogenic compounds represents a practical and efficient approach in the preparation of enantio-enriched P-stereogenic compounds, especially when racemic forms are readily available whereas enantiopure forms are not. Compared to desymmetrization strategies, catalytic kinetic resolution protocols are less developed for accessing P-stereogenic chirality.

In 2009, Tan and co-workers reported a pioneering work, in which a chiral guanidinium salt (**C9**)-catalyzed phospha-Mannich reaction of imine (**39**) with secondary phosphine oxides or H-phosphinates producing P-stereogenic α-amino phosphinates was reported (Fu et al., 2009). With the use of 3.0 equiv. racemic H-phosphinates (*rac*-**38**) as nucleophiles, the reaction realized an enantioselective construction of P-stereogenic α-amino phosphinates (**40**) with good to excellent enantio- and diastereo-selectivities as representative examples, as shown in Figure 2H and Supplementary Scheme S9a. When using 1.0 equiv. H-phosphinate *rac*-**38a** as a nucleophile, the Mannich reaction with imine **39a** resulted in the kinetic resolution of H-phosphinate *rac*-**38a**, producing enantio-enriched H-phosphinate (*S*)-**38a** (32% yield; 87% ee) and α-amino phosphinate **40f** (*syn* and *anti*, 52% yield, 1.6:1 dr, and 50% ee) (Figure 2H; Supplementary Scheme S9b).

In 2012, Zhang and co-workers reported chiral bicyclic imidazole **C10**-catalyzed kinetic resolution of phosphorochloridothioate to generate P-stereogenic phosphoramides (Liu et al., 2012). As shown in Figure 2I and Supplementary Scheme S10, under the catalytic system, the reaction of *O*-ethyl *S*-propyl phosphorochloridothioate (EPPC, **41**) with amides or amines gave rise to the corresponding phosphoramides with P-chirality, although both the conversion rate and enantioselectivity were poor. It was speculated that the catalytic process involved (1) the selective formation of two diastereoisomers of ammonium intermediates *via* the reaction of phosphoryl chloride **41** and the chiral catalyst **C10**, and (2) diastereoselective attack of the amino compound **42** on these two active intermediates (**44** and *epi*-**44**) producing the optically enriched product **43**, with the release of the catalyst. The second step was rate-determining, and both steps contributed to the enantioselectivity of the products.

Secondary phosphine oxides are electron-rich in the “P” center and are usually used as nucleophiles and ligands in synthetic chemistry (Shaikh et al., 2012). In 2020, Zhang and co-workers reported Le-Phos (**C11**)-catalyzed kinetic resolution of secondary phosphine oxides (*rac*-**45**) *via* the asymmetric allylation reaction with Morita–Baylis–Hillman carbonates (Figure 2J; Supplementary Scheme S11) (Qiu et al., 2020). A variety of optically pure secondary phosphine oxides (*R*)-**45** and tertiary P-stereogenic phosphine oxides (**47**) were prepared utilizing this method with good yields and high enantioselectivities. Moreover, this reaction could be performed on a gram-scale without the loss of enantioselectivity. The resulting P-stereogenic products were suitable for further transformations to obtain optimal P-stereogenic catalysts and ligands (Supplementary Scheme S11b).

## Asymmetric dynamic kinetic resolution strategies

Although the kinetic resolution strategy can provide optically pure products, it is limited to a maximum theoretical yield of 50%. Thus, dynamic kinetic resolution (DKR) has drawn more attention for preparing P-stereogenic phosphorus compounds as the yield can be theoretically increased to as much as 100%.

Phosphoramidate prodrugs (mostly containing P-stereogenic centers) are a key component of pronucleotide (ProTide) therapies for the treatment of viral diseases and cancer. In 2017, DiRocco and co-workers reported a bicyclic imidazole-derived multifunctional catalyst (**C12**) and applied it to the synthesis of ProTide MK-3682 (**54a**), which is in late-stage clinical trials for the treatment of HCV disease (DiRocco et al., 2017). As shown in Figure 2K and Supplementary Scheme S12, **C12** mimicked the complex function of enzyme catalysis *via* a distinctive activation mode. In the catalytic system, chlorophosphoramidate (**52**) is in rapid equilibrium with activated species **55a** and **55b**, and P–O bond formation is the turnover-limiting step. Despite the fact that the catalyst was designed for preparing MK-3682 (**54a**), the catalytic system was suitable for asymmetric phosphoramidation of multiple nucleoside analogs.

Dynamic kinetic asymmetric transformation (DyKAT) showed its potential in the synthesis of the anti-SARS-CoV-2 drug remdesivir. Shortly after the breakout of COVID-19, Wang et al (2020b) responded rapidly to report a chiral bicyclic imidazole **(C13**)-catalyzed coupling of P-racemic phosphoryl chloride (**56**), with a protected nucleoside GS-441524 (**57**), which promoted asymmetric access to the P-stereogenic structure of remdesivir (Figure 2L; Supplementary Scheme S13). This process involves a smoothly dynamic kinetic asymmetric transformation (DyKAT) with high reactivity and excellent stereoselectivity (96% conv., 22:1 *S*_P_:*R*_P_).

Gannedi et al. (2021) have also reported chiral bicyclic imidazole (**C14**)-catalyzed asymmetric (*S*)-P-phosphoramidation for the synthesis of remdesivir (Figure 2M; Supplementary Scheme S14). Under optimized reaction conditions, the desired (*S*)-P-phosphoramidate **60** was obtained with 73% yield and a 99.4:0.6 dr ratio (after recrystallization), when 20 mol% of the catalyst **C14** was employed as a catalyst. Furthermore, a 10-g-scale one-pot synthesis *via* a combination of (*S*)-P-phosphoramidation and protecting group removal, followed by one-step recrystallization, produced remdesivir with a 70% yield and 99.3:0.7 dr.

Additionally, chiral bicyclic imidazole-catalyzed asymmetric P-phosphoramidation was applied in the total synthesis of the antiviral agent uprifosbuvir by Klapars et al. (2021). A five-step synthesis of uprifosbuvir with 50% overall yield, from readily available uridine (**61**), was reported (Figure 2N; Supplementary Scheme S15). The synthetic route features the following: (1) complexation-driven selective acyl migration/oxidation; (2) BSA-mediated cyclization to anhydrouridine; (3) hydrochlorination using FeCl_3_/TMDSO; and (4) dynamic stereoselective P-phosphoramidation. The key stereoselective P-phosphoramidation of alcohol **65** with chlorophosphoramidate **52** employed only 3 mol% loading of the bicyclic imidazole catalyst **C15**, providing uprifosbuvir (**66**) with a ratio of 97:3 dr, 94% assay yield, and 88% isolated yield of uprifosbuvir after crystallization. This asymmetric P-phosphoramidation-based route achieved a 50-fold improvement in the overall yield of uprifosbuvir over the previous manufacturing process.

In addition to the aforementioned bicyclic imidazole-type catalysts, chiral phosphoric acid has also emerged as a powerful catalyst for constructing P-stereogenic molecules. Recently, Featherston et al. (2021) demonstrated the chiral phosphoric acid (CPA)-catalyzed formation of stereogenic phosphorous centers during phosphoramidite transfer (Figure 2O; Supplementary Scheme S16). Both peptide-embedded phosphothreonine-derived CPAs (**C18**) and C2-symmetric BINOL-derived CPAs (**C16–C17**) were investigated in the study, which gave rise to unprecedented levels of diastereodivergence, enabling access to either phosphite diastereomers. Diastereodivergent catalysis can be applied to other nucleobase pairs, demonstrating the broad fundamental significance and utility.

## Conclusion and outlook

Organocatalytic methods to access P-stereogenic scaffolds have made great progress during the last decade. Strategies based on desymmetrization and (dynamic) kinetic resolution have attracted most of the work and are still mainstreamed in the development. Multiple catalytic systems were developed, with numerous optically enriched P-stereogenic molecules prepared. Nevertheless, investigations on new catalytic modes and diversified substrates are still highly demanded. In the coming years, we expect to see an expansion in new-type organocatalytic methodologies and applications of these strategies in the creation of medicines, natural products, and other functional P-stereogenic molecules.
