# Supplementary material for: Organocatalytic asymmetric synthesis of P-stereogenic molecules
Source: Front Chem. 2023 Feb 16;11:1132025. doi: 10.3389/fchem.2023.1132025 (PMC9978094; doi:10.3389/fchem.2023.1132025)
Supplement: Supplementary file 1 [file DataSheet1.pdf]

Supplementary Materials

Organocatalytic asymmetric synthesis of P-stereogenic molecules

Junyang Liu[1,2†], Hang Chen[3†], Min Wang[1], Wangjin He[1], Jia-Lei Yan[1*]

[1]Innovation Center of Marine Biotechnology and Pharmaceuticals, School of Biotechnology and Health Sciences, Wuyi University, Jiangmen, China
State Key Laboratory of Chemical Oncogenomics, Key Laboratory of Chemical Genomics,
[2]Peking University Shenzhen Graduate School, Shenzhen, China
[3]Division of Chemistry & Biological Chemistry, School of Chemistry, Chemical Engineering and Biotechnology, Nanyang Technological University, Singapore, Singapore

The Supplementary Schemes are shown below:

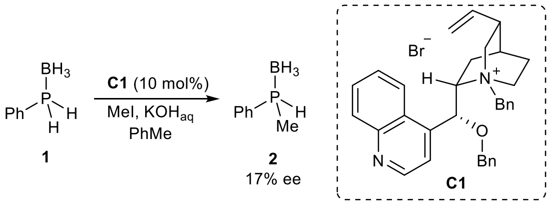

SCHEME S1. Synthesis of P-stereogenic phosphine borane complexes by phase-transfer catalysis.

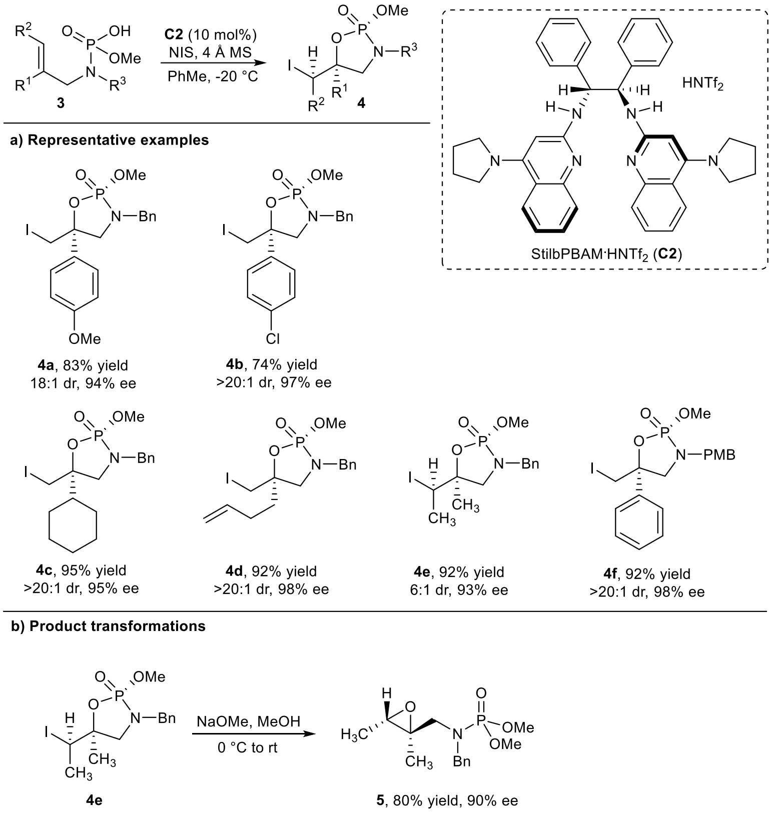

SCHEME S2. Chiral Brønsted acid catalyzed diastereo- and enantioselective iodocyclization

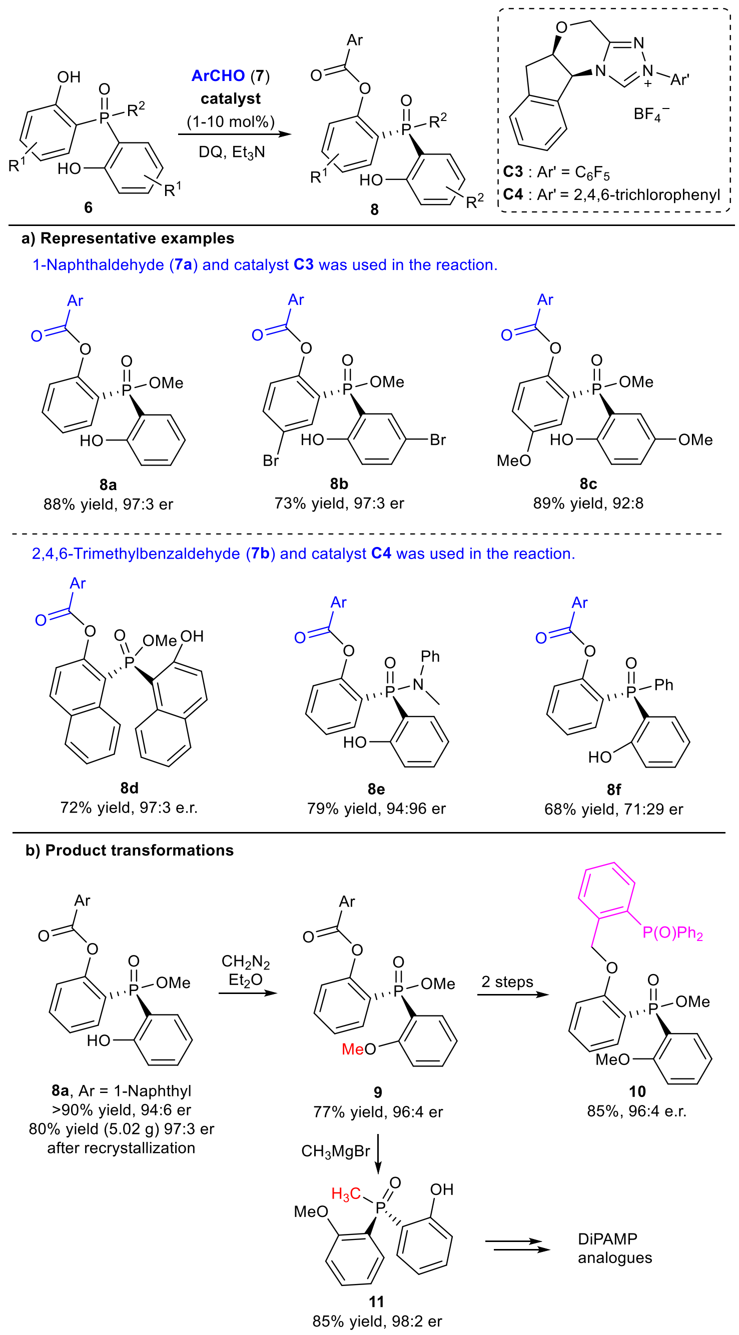

SCHEME S3. NHC catalyzed desymmetrization of prochiral bis(2-hydroxyphenyl) phosphinates.

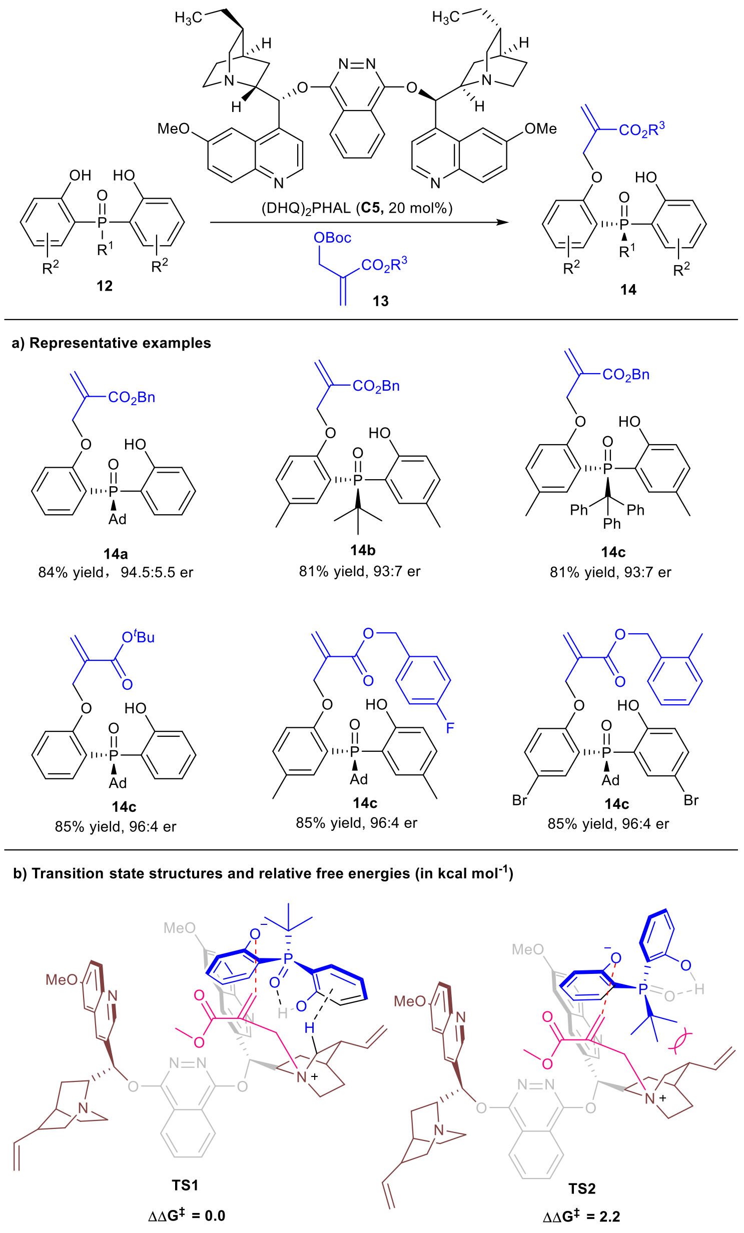

SCHEME S4. Desymmetric allylation reaction of bis(2-hydroxyphenyl)phosphine oxides with Morita-Baylis-Hillman carbonate. Reproduced from [G. Yang, Y. Li, X. Li and J. Cheng, *Chem. Sci.*, 2019, **10**, 4322. **DOI:** 10.1039/C8SC05439H] with permission from the Royal Society of Chemistry.

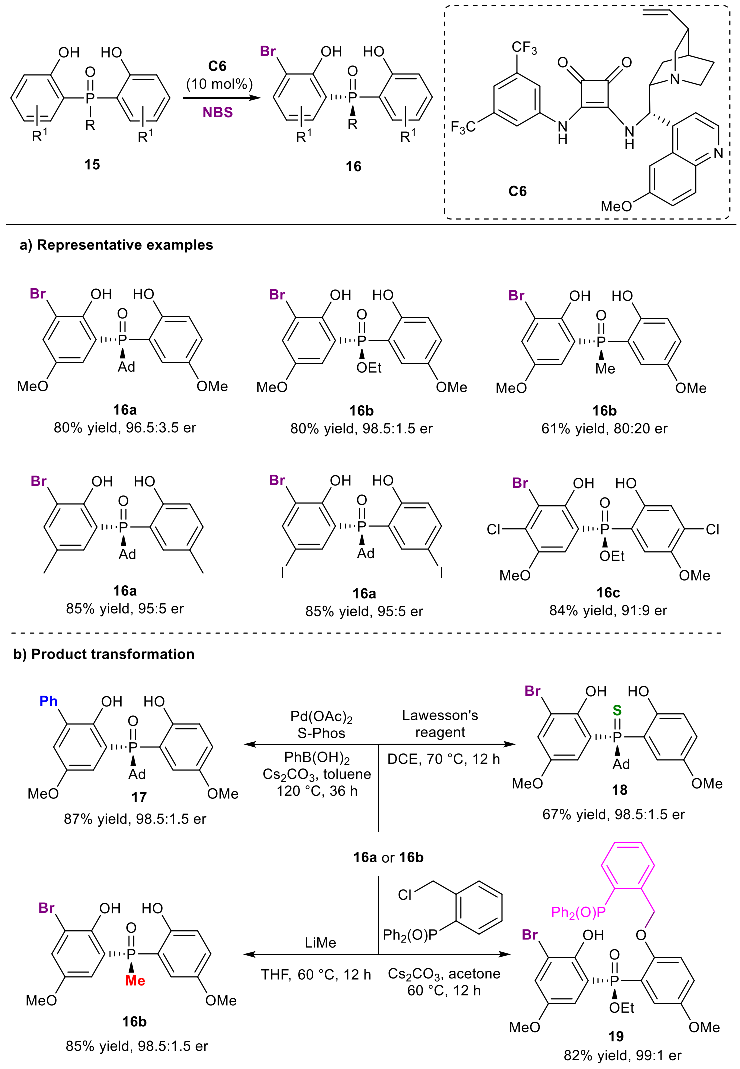

SCHEME S5. Desymmetrization of bisphenol phosphine oxides via ortho-selective mono-bromination.

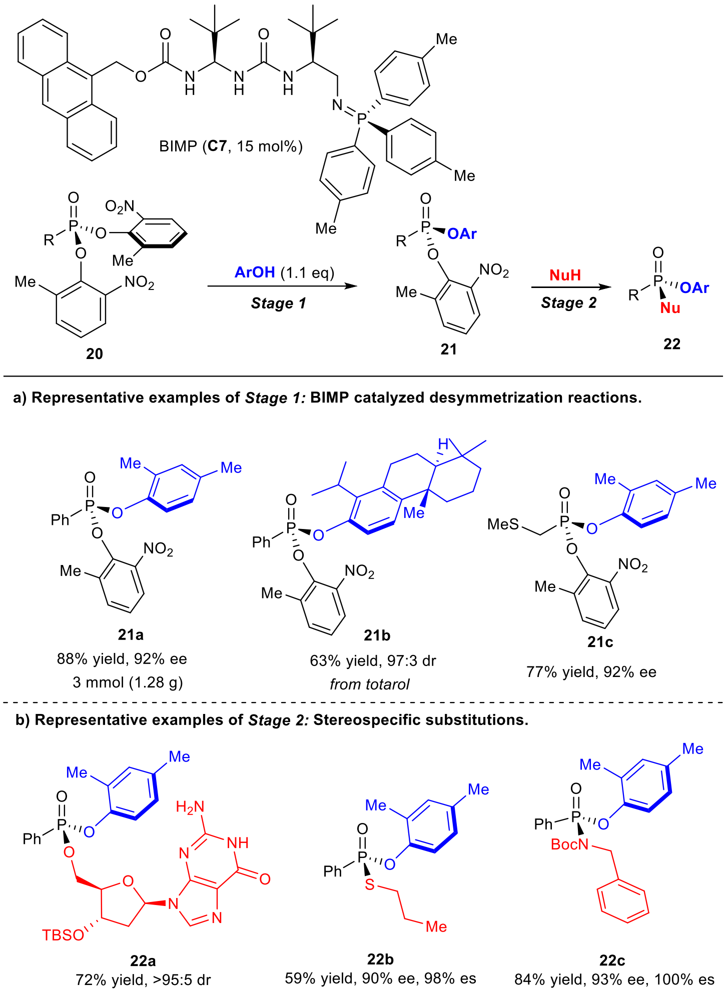

SCHEME S6. BIMP catalyzed desymmetrization of phosphonate esters.

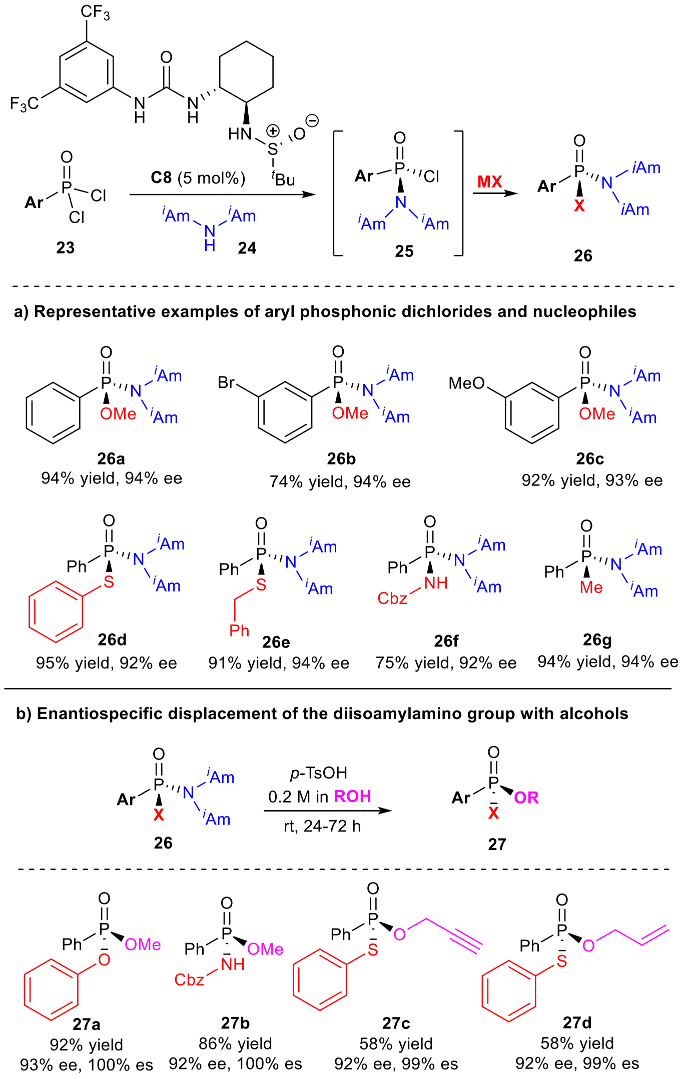

SCHEME S7. Hydrogen-bond-donor catalyzed desymmetrization of aryl phosphonic dichloride to access chiral P(V) compounds.

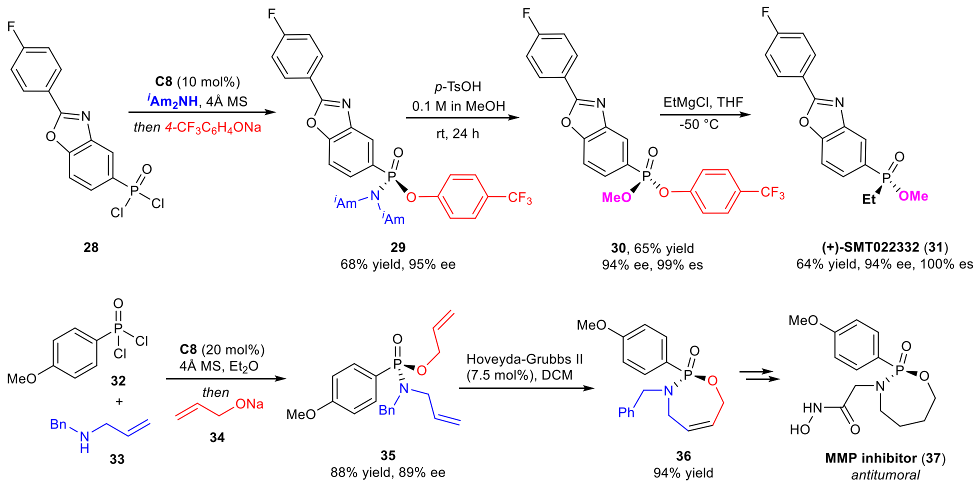

SCHEME S8. Synthesis of P(V)-stereogenic bioactive molecules via hydrogen-bond-donor catalyzed desymmetrization strategies.

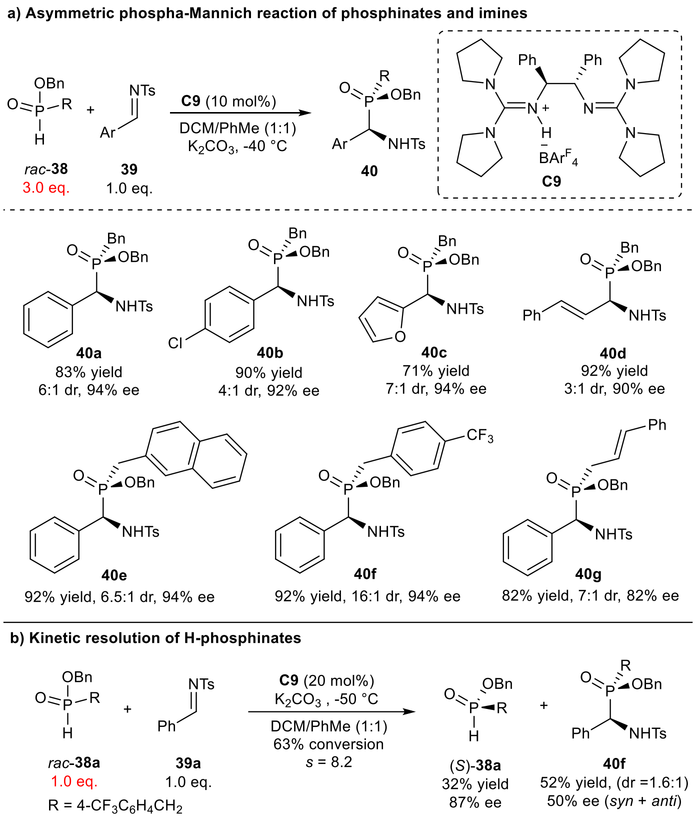

SCHEME S9. Chiral guanidinium salt catalyzed enantioselective phospha-Mannich reactions

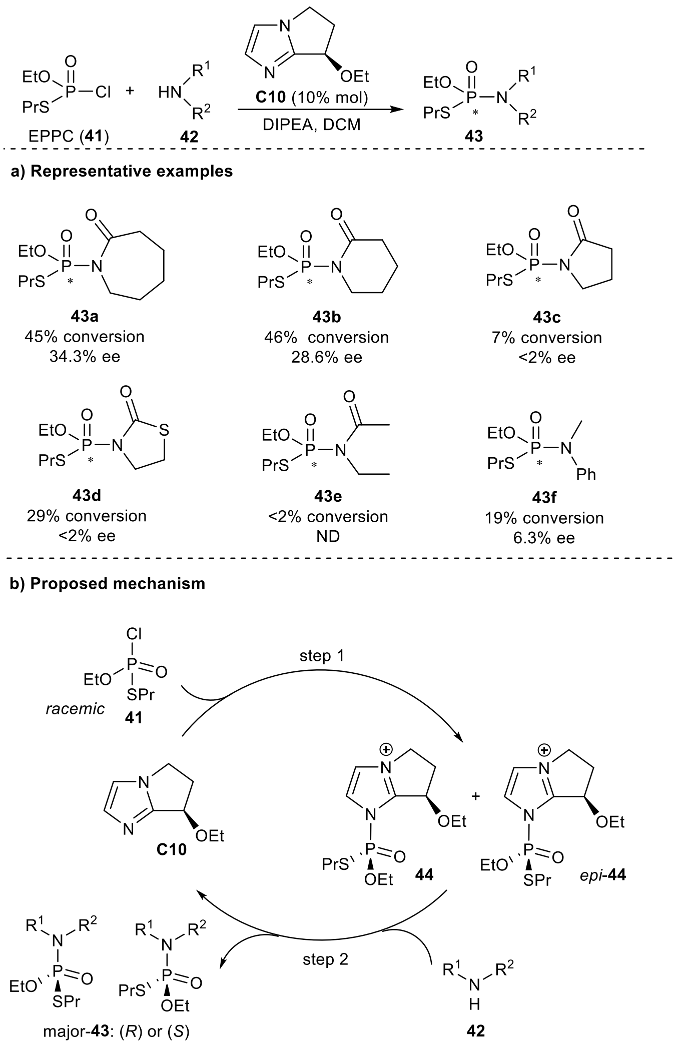

SCHEME S10. Chiral bicyclic imidazole catalyzed kinetic resolution of phosphorochloridothioate

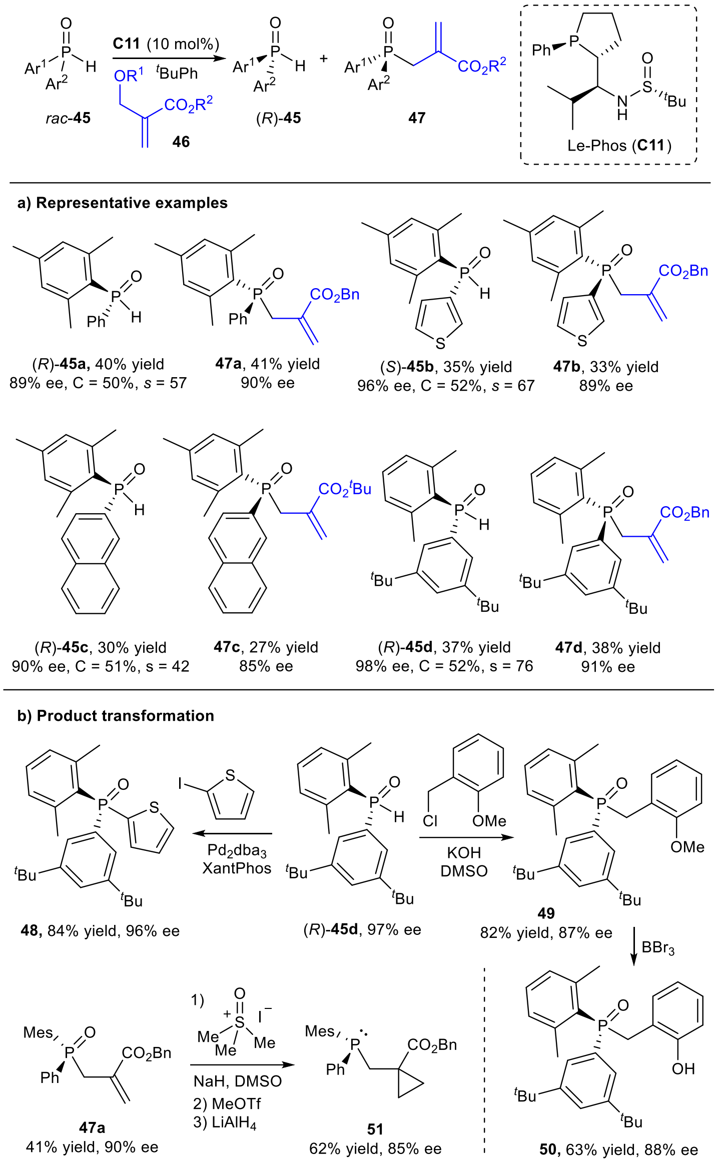

SCHEME S11. Le-Phos-catalyzed kinetic resolution of secondary phosphine oxides

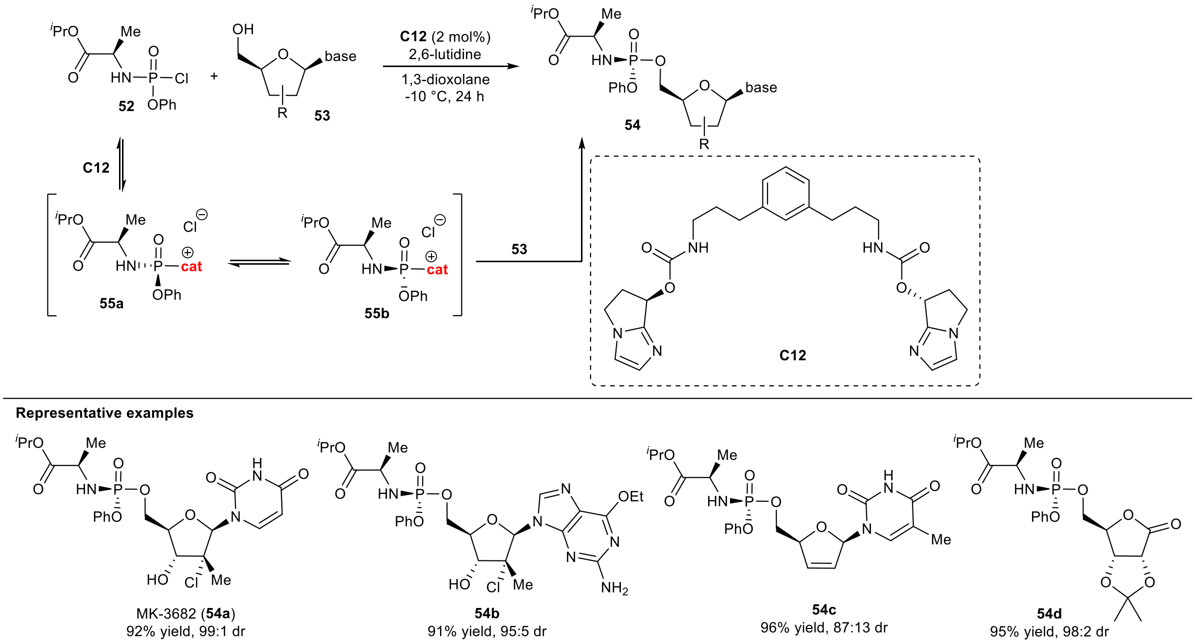

SCHEME S12. Catalytic stereoselective phosphoramidation of nucleosides.

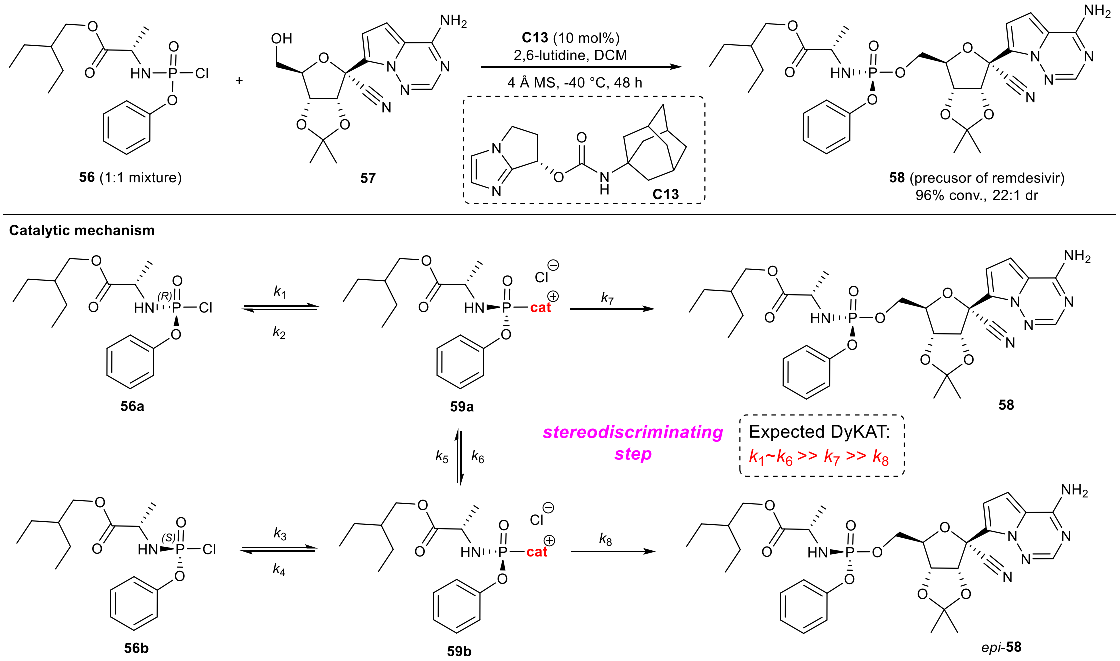

SCHEME S13. Chiral bicyclic imidazole catalyzed asymmetric synthesis of remdesivir.

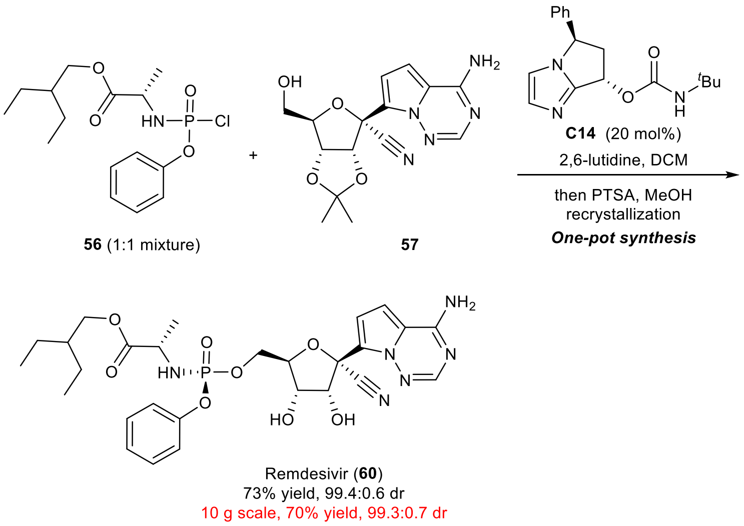

SCHEME S14. Synthesis of remdesivir through a one-pot organocatalyzed asymmetric (*S*)-P-phosphoramidation

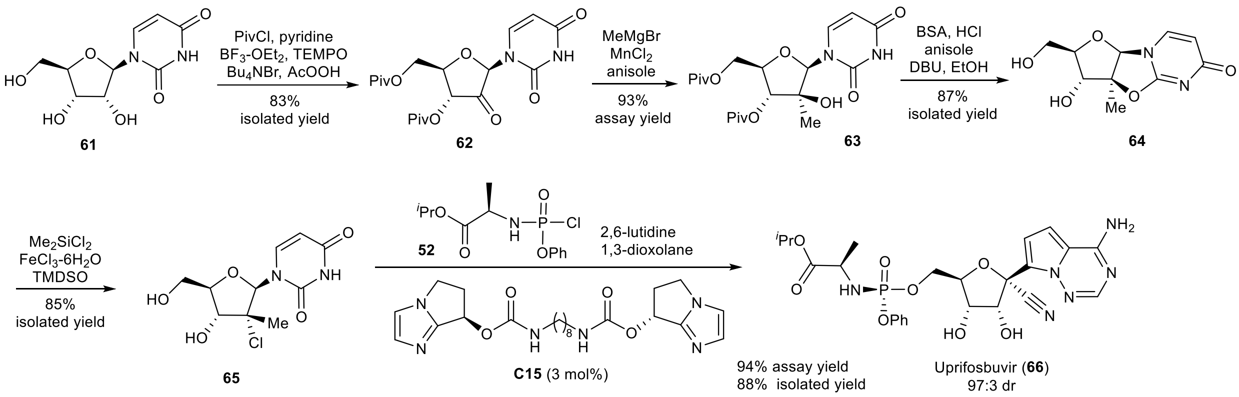

SCHEME S15. Asymmetric synthesis of uprifosbuvir

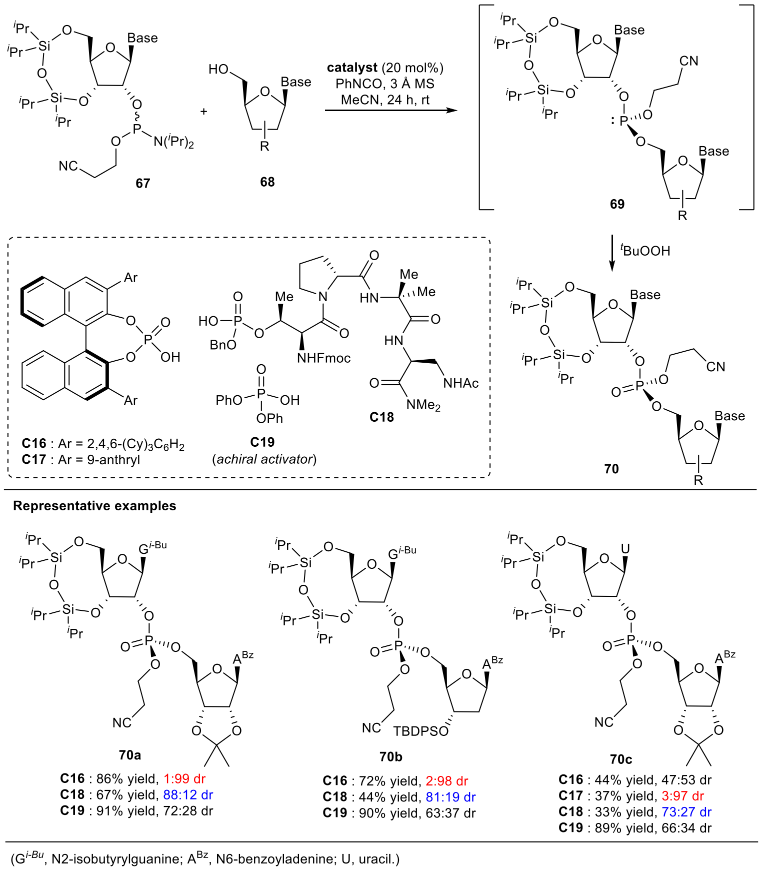

SCHEME S16. Chiral phosphoric acid catalyzed asymmetric and stereodivergent synthesis of oligonucleotide.
